# Intersections between disability, masculinities, and violence: experiences and insights from men with physical disabilities from three African countries

**DOI:** 10.1186/s12889-022-13137-5

**Published:** 2022-04-10

**Authors:** Yandisa Sikweyiya, Erin Stern, Jill Hanass-Hancock, Ingrid van der Heijden, Henri Myrttinen, Adolphina Addoley Addo-Lartey, Kristin Dunkle

**Affiliations:** 1 Gender and Health Research Unit, South African Medical Research Council, Pretoria, South Africa; 2 School of Public Health, University of the Witwatersrand, Johannesburg, South Africa; 3 Gender Violence and Health Centre at the London School of Hygiene and Tropical Medicine, London, UK; 4 School of Health Sciences, University of KwaZulu-Natal, Durban, South Africa; 5 Independend consultant and researcher, Amsterdam, Netherlands; 6 International Alert, London, UK; 7 Department of Epidemiology and Disease Control, School of Public Health, University of Ghana, Accra, Ghana

**Keywords:** Disability, Masculinities, Violence, Emotional violence, Violence prevention programming

## Abstract

**Background:**

Gender-transformative work in the Global South often focuses on transforming ‘toxic masculinities’ to prevent intimate partner violence (IPV), but there has been little research on whether and how constructions of masculinities by men with disabilities shape their experiences and perpetration of violence.

**Methods:**

We used repeated in-depth interviews and content analysis to understand whether and how physical disability intersects with the construction of masculinities and experience/perpetration of violence among 15 adult men with physical disabilities participating in interventions to prevent IPV in Ghana, Rwanda, and South Africa.

**Results:**

Societal expectations and participants’ aspirations around masculinity impacted their vulnerability to violence mainly by men without disabilities. Participants reported experiences of disrespect and social exclusion in their communities and felt incapable of protecting themselves when being violated. Most participants felt they were not providing for their families and perceived themselves as having lost decision-making and positions of power in their homes. They expressed their disappointment with having reduced stamina, virility, and sexual prowess in intimate partnerships as a result of their disability. While participants reported that they could not attain key markers of idealized masculinity, placed upon and often internalized by themselves, they longed to achieve these markers to facilitate their inclusion and acceptance in their communities.

**Conclusions:**

Programmers addressing violence need to engage with men with physical disabilities and consider the intersectionality of masculinities and disability, how these reinforce patriarchal norms and how men with disabilities can be included and enabled to overcome their conflict between disability and masculinities.

## Background

Intimate partner violence (IPV) prevention in Africa often includes a focus on transforming harmful masculine norms and behaviours, such as men’s aggression, sense of entitlement to women and dominance over their families, controlling sexual behaviours, and substance abuse that influences their use of violence against women [1–3].

However, these gender-transformative approaches to IPV prevention rarely consider or directly address masculinity as embodied or experienced by men with disabilities, and there is little research on whether and how constructions and performance of masculine positions and behaviours by men with disabilities shape their experiences and perpetration of violence. Scholarship on masculinity and disability largely reflects data from the Global North, and rarely features the experiences and insights of men with disabilities in the Global South [4].

We premised this study within concepts of gender and draw specifically from Connell’s [5] hegemonic masculinity and Crenshaw’s [6] intersectionality theoretical concepts. Connell [5] proposed the concept of hegemonic masculinity to explain the reasons and means by which gender inequality is maintained. Through the concept, Connell [7] extended feminist theory, which highlighted inequality *between* men and women, by further illustrating inequality *within* the category of men. Regarding hegemonic masculinity, she argued that in any given setting multiple masculinities are circulating, yet among these exist a communal cultural model of masculinity that is perceived as an ideal and superior to the other masculinities [8].

In the hierarchy of masculinities, the dominance maintained by the hegemonic masculinity is obtained through a social consensus, instead of violent repression of the subordinate masculinities [9], even though violence is always retained as an option. The concept of hegemonic masculinity has been extensively employed in studies on men and masculinities in the sub-Saharan region as a theoretical framework to highlight the hierarchy and multiplicity of masculinities among men [10–13].

Intersectionality, first formulated by Crenshaw [6], is concerned with highlighting and addressing variations within what is deemed collective identities. Crenshaw asserted that, in their appreciation of social relations and representation of oppressed groups, social movements tend to focus on one aspect of individuals’ identities. Within such unidimensional views of identities, significant intersections of identities that exacerbate oppression for some are often neglected. Likewise, identities that alleviate oppression for others are obscured [6]. The intersectionality lens has been applied, albeit implicitly, in some studies of men and masculinities in the sub-Saharan region. Such studies often concern the contradiction between gendered privilege and race and class-based historical marginalisation among the majority of black men [14–16]. However, there is a paucity of research that has used an intersectionality lens in understanding the lived experiences of and constructions of masculinities by men with disabilities in the sub-Saharan region.

Disability itself is “an evolving concept” that results “from the interaction between persons with impairments and attitudinal and environmental barriers, that hinders their full and effective participation in society on an equal basis with others” (CRPD 2006). Hence while impairment is the loss of physical, mental, or sensory functioning, disability is a social construct. Discrimination based on impairment status hinders the persons with disabilities to achieve their set ambitions, capabilities, and in so doing participation in society.

Capabilities are practical opportunities that a person utilises based on their ambitions and decisions in life (e.g., to go out of the house, engage in work or leisure, being a father or husband). Framed through the Capability approach “disability occurs when an individual is deprived of practical opportunities as a result of an impairment” [17]. Using this approach, disability is seen as caused by a combination of a) the impairment itself, b) the resources available to a particular person and c) environmental and attitudinal barriers [17]. For instance, a man with physical disabilities can be deprived of practical opportunities as a result of the loss of physical functioning (e.g. a person cannot walk out of the house and needs a wheelchair), higher levels of poverty (e.g. decreased income-generating opportunities and higher disability-related costs) and environmental barriers based on physical, economic, social, political or cultural aspects (e.g. public streets and facilities are not wheelchair accessible, men with disability are not perceived as family providers). The experience of disability also intersects with other characteristics such as gender, race, or economic status. Hence under similar conditions men and women with disabilities have different capabilities (practical opportunities) and experience different types of opportunities and deprivations based on their gender and disability.

Dominant social constructions of disability “as weak and dependent” versus ‘being strong and independent as a man’ often result in a “dilemma” [18]. Disability is often conceptualized as an “embodied lack” [19], and for men with disabilities, this limits their ability to achieve dominant masculine expectations of strength, independence, and self-reliance [18].

Men with disabilities tend to be stigmatised by society and often socially marginalized due to their “disabled” body [20]. An argument has also been made that men with disabilities, as with non-disabled men, tend to identify with hegemonic ideals of masculinity such as physical strength, independence, and bravado, which places them in conflict with the perceptions of their body as ‘weak and not able’ [21]. Yet, Addlakha [22] contends that men with physical disabilities may be particularly stigmatized because of the visibility of impairments (their “discrediting attributes”).

While masculinities are fluid and intersect with context and identity, there often are certain attributes, in Africa as elsewhere, that are commonly associated with successful manhood. However, how men with disabilities in the Global South negotiate their capabilities (practical opportunities) in their social contexts and how their perceptions and environments may impact the experience or perpetration of violence and abuse is currently not well understood. In this paper, we explored whether and how physical disability intersects with the construction of masculinities and men’s experiences or perpetration of violence.

### Context of the study

The data presented in this paper were drawn from a multi-country qualitative study focusing on disabilities and violence that assessed the inclusion of people with disabilities in IPV prevention programmes, in three countries i.e. COMBAT in Ghana, CHANGE in South Africa, and Indashyikirwa in Rwanda. These programmes were evaluated as part of the What Works to Prevent Violence against Women and Girls Global Programme.^1^ The qualitative study was designed to supplement findings from the parent studies (i.e. COMBAT, CHANGE, and Indashyikirwa evaluations) and explored men with physical disabilities’ perspectives on gender, disability, and violence, and appraised whether and how the three programmes included individuals with disabilities and met their disability-related needs [23].

Participants were recruited from rural and peri-urban areas in Ghana, peri-urban and rural communities in Rwanda, and an urban informal settlement in South Africa. In all three countries, the participants came from communities with relatively similar socio-economic backgrounds, with poverty burdening many households. In Ghana, the study was conducted in the Central Region which has an unemployment rate of 8.0% which is 2.4% lower than the national average. In this region, agriculture is the main occupation, and it employs more than two-thirds of the workforce in many districts. Fishing is concentrated mainly along the coast, whereas cocoa and oil palm production is concentrated inland. In the Central Region of Ghana, a recent study evaluating the COMBAT programme showed that one in seven men had not worked in the past 12 months, with about two-thirds of the men being moderately or severely food insecure [24]. In Rwanda, the Indashyikirwa programme recruited couples and community activists drawn from CARE Rwanda’s village savings and loans associations, which target households from the most deprived socio-economic backgrounds [25]. The programme was mainly implemented in rural areas, whereby the livelihoods of many community members depends on subsistence farming. The couples curriculum and subsequent community activism recruited women and their male partners from CARE Rwanda’s micro-finance village savings and loans associations (VSLAs) which aim to benefit households that are particularly deprived socio-economically. In South Africa, participants lived in a densely populated peri-urban settlement. While most residents lived in government-subsidised houses, some lived in informal tin shacks. In this settlement, electricity and water are available for large parts of it, yet there are parts of the settlement where there is lack of access to basic services such as running water, sewer and rubbish removal. Moreover, the settlement is characterized by high poverty and unemployment rates, and high levels of violence and ill-health [26].

## Methods

### Study design, sampling, and participants

An explorative qualitative study employing in-depth interviews (IDIs) was conducted to gain narrative insights into the lived experiences of violence among men with physical disabilities in communities in Ghana, Rwanda, and South Africa. This data is drawn from a wider study that qualitatively interviewed both men and women living with disabilities across four of the What Works to Prevent Violence against Women and Girls interventions [23].

A total of 15 adult men with a range of physical disabilities participated in the study. A convenience sampling approach was used to enrol participants in the study. Programme staff were primed to identify men with disabilities either among programme participants (Ghana and Rwanda) or residing in the programme catchment area (South Africa). Participants were aged 21–63 years old with a mean age of 40 (SD 14.79673), and were mostly educated up to grade 10 (see Table 1). They were mainly reliant on seasonal agricultural labour in Rwanda and Ghana, and informal work in the urban township in South Africa. Some participants reported challenges with securing employment because of the inaccessibility of environments. All men in the study reported at least one former or current intimate relationship. Only a few (*n* = 3) were born with a physical disability (i.e., congenital disability) whereas a majority (*n* = 12) had acquired a physical disability later in life as a result of a health event or injury. These participants’ **characteristics** show that most men in our sample had a lived experience as ‘able-bodied’ boys or men and therefore experienced ‘loss’ of functioning as adult men.

**Table 1:** Demographic information of participants (all heterosexual men)

*N* = 15	N	*N* = 15	N
**Age**		**Relationship status**	
21–25	2	Married	9
26–30	3	Single	4
31–40	3	Divorced	1
41–50	4	Cohabiting	1
51–63	3		
**Impairments**		**Employment**	
Congenital	3	Unemployed	8
Acquired	12	Informal employment	4
**Children**		Formal employment	3
Yes	11	**Education**	
No	4	None/dropped out early	4
		Lower Secondary (7th–9th Grade, 10–12 years old)	3
		Upper Secondary (10-12th Grade, 12–18 years)	3
		Tertiary Diploma/Degree	5

### Data collection

Repeat in-depth interviews (IDIs) were conducted with these 15 men with physical disabilities in Rwanda (*N* = 6); South Africa (*N* = 4); and Ghana (*N* = 5). Semi-structured interview guides were used to explore participants’ experiences of disability and violence both within intimate partnerships and in other community and relational contexts. Interview topics included gender norms in their communities, discrimination based on disability, community safety, and intimate partnerships. Questions on violence included participants’ experiences of victimization and perpetration of violence, including questions probing for disability-specific violence (for example being denied care or help, interference with assistive devices, or control over money by caregivers).

Participants were recruited and interviewed either once or twice until the study coordinator (IVDH) was confident that data saturation had been reached, as similar themes were being elicited from the data to answer the exploratory areas of interest [27]. In general, initial interviews were used to build rapport, and the second interview explored in-depth sensitive issues that were raised in the previous interviews. Questions about violence were asked once the interviewers felt rapport had been established with participants, either at the latter part of the first interview or during the second interview.

Socio-demographic information was collected during the interviews detailing age, type, and nature of physical impairment and when it was acquired, educational level, marital status, number of children, and employment status. Each interview lasted approximately 1.5 h. All interviews were conducted in participants’ preferred language, audio recorded with the consent of the participant, and translated and transcribed verbatim into English by in-country professional translators that had experience translating qualitative transcripts into English.

Research coordinators from all three countries attended disability-sensitive research training conducted by (IVDH). In Rwanda, interviews were conducted by an experienced female researcher without disability. In Ghana and South Africa, male interviewers, without disability, and external to the programmes were employed and also received disability-sensitive training research before conducting the interviews.

### Data analysis

Latent content analysis [28] was used to analyze the 28 transcribed interviews conducted with the 15 men using Atlas-Ti 8. The data were analysed inductively. First, the authors (YS, ES, JHH and KD) read the transcripts repeatedly to familiarize themselves with the content of the transcripts. Next, they created broad codes which were relatively similar to the questions in the interview guide. Thereafter, the text which appeared to fit together was grouped under a specific code. In addition, the authors explored the data and identified open codes as emerging from the data. These open codes were also clustered under defined themes. Last, the authors explored the relationships between the themes and interpreted what they saw emerging. Authors (IVDH, ES, AAL, YS and KD) from each of the settings who knew the interventions and contexts well were involved in interpreting the coded data for language, cultural or contextual factors.

### Ethical considerations

Ethical approval for the study was obtained from the South African Medical Research Council’s Ethics Committee (EC022–7/2016) and the Institutional Review Board at the Noguchi Memorial Institute for Medical Research, University of Ghana, (NMIMR-IRB CPN 102/16–17) and the Rwandan National Ethics Committee (RNEC) (REF: 340/RNEC/2015) and the National Institute of Statistics Rwanda (REF:0738/2015/10/NISR).

Before conducting the IDIs, the interviewers reviewed the participant information and consent form with the prospective participants. The discussion included the rights of participants and the risks and benefits of participating in the study. Written informed consent for study participation and publication of the study findings was obtained from all participants. All identifying information of participants has been removed for the presentation of the findings.

Participants were reimbursed their travel costs to interview sites where applicable. Participants also received a cash incentive of about $5 as a gesture of the investigators’ appreciation for participating in this study. In some sites, refreshments were provided after each interview in the form of bottled water, and a healthy snack.

## Results

Four main themes around men’s constructions of masculinity and their vulnerability to violence emerged during data analysis: (dis) respect and social exclusion in their communities, protecting themselves and finding recourse to violence and injustice, financial provision and decision-making power in the home, and showing stamina, virility, and sexual prowess in intimate partnerships.

### (dis) respect, humiliation, and social exclusion

Participants reported experiencing marginalization in their communities, with many emphasizing that men without disabilities were not subjected to the same marginalization. Maintaining the persona of an in-control, able and strong man was important to most participants in all three settings. Yet, owing to mobility challenges, lack of assistive devices, physical pain, self-stigma, and perceived social stigma, the majority of participants experienced isolation and exclusion from community activities, which they experienced as impacting their social standing and respect as men. Like Participant 1, many participants felt their exclusion in their communities compounded their vulnerability, which made them perceive an enhanced risk of suffering community violence: “*If you show weakness, you will be less of a man, and you will be under threat*”. (Ghana, 63 years, Participant 1).

The type and severity of impairments was seen as related to the degree of exclusion. For instance, men with more severe disabilities, which limited their mobility, reported that they had very few opportunities to participate in their community activities. Participant 2’s extract indicates this: ‘*It is rare for me to be with friends. Sometimes I just sit here or at the house. I watch TV. I do not socialise often*’. (South Africa, 23 years, Participant 2).

Some participants reported a lack of suitable and functioning assistive devices which curtailed their freedom of independent mobility, and which kept them from joining in communal activities:

My wheelchair gets weary [damaged by the environmental conditions where I live … ] So it would help me if I had an electronic wheelchair so that I can move better. Sometimes my hands get tired. (South Africa, 29, Participant 3)

Participants in all three settings described how men with disabilities often experienced ridicule from community members. Participant 4 from Ghana narrated how he often got shouted at and told to keep quiet when he attempted to speak during community meetings, attributing this treatment to his disability:

We [men with a disability] are not able to join communal activities as others, but some of the community members mock you when they see you, to let you feel that you cannot join them during communal activities because of your disability. They often shout at us to keep quiet and sit down when we attempt to make a suggestion during community general meetings. (Ghana, 45 years, Participant 4)

Similar sentiments were shared by Participant 5 from South Africa who described being excluded from engaging in male activities, like playing pool and being exposed to verbal abuse and name calling. He said:

They [people without disability] think they are better because they are not disabled. They do not consider me as a human being … They make nasty comments towards us. I did not wake up wishing to be this way. Some would call me while I am sitting on the corner by the street, shout at me and say “hey, you with crutches, what are you doing there? Why don’t you stay at home?” Or maybe while playing pool, they would ask me what I am doing there and say I am not wanted there … People call me names here too and it hurts me. I cannot walk without crutches, but mentally we are the same. But we do not see it the same way. (South Africa, 29, Participant 5)

The ridicule experienced by the men shaped their self-stigma and shame, making them feel less respected by other men and fellow community members. The data suggest that the fear of potential embarassment and shame plunged them deeper into isolation:

I often distance myself from such gatherings … I cannot go there and walk about like other ordinary people … My fear is that due to my impairment, my leg may twist and I may fall in the presence of [strangers] who do not know my situation, they might think I am drunk, that is why I feel ashamed to join community gatherings. (Ghana, 45 years, Participant 4)

Their rejection and/or exclusion from community activities made most participants feel powerless and disrespected. This is evidenced by some participants’ assertion that in their communities, men without disabilties did not take their voices seriously. This, according to these participants, occurred despite the fact that men are typically given priority in decision-making in their communities:

I got the understanding that if I dare to contribute to the discussion, I will be shut down and forced to sit, so I should just keep quiet, sit, observe and listen to their considerations. (Ghana, 45 years, Participant 4)

Participant 4 further related how men with disabilities experienced diminished respect in their communities, especially around their suitability for marriage:

We are accorded no respect. The respect we have is in a decreasing manner and I do not know if there will be a change in how people see us [men with a disability]. If we have some respect, why will the relative of the woman I love tell me they will not allow me to marry their daughter because I am a disabled man? We are always belittled (Ghana, 45 years, Participant 4).

These narratives suggest that across all three settings, the masculinities of men with disabilities were generally considered subordinate to those of men without disabilities, and this was chiefly underscored by the latter who reportedly treated men with disabilities with disdain. For these men, many opportunities to participate (their capabilities) in society were restricted.

However, this was not experienced by all men interviewed. Participant 6 from Rwanda explained how he had never faced ridicule or exclusion in his village. He was able to provide for his family and that this opportunity (capability) counteracted the potential discrimination and isolation he could face because of his disability. Participant 6’s narrative suggests that he had achieved some key markers of masculine success i.e., he was independent, married, able to economically provide for his family, and had a good relationship with the local leadership:

The reason behind [being respected] is that I manage to provide for my family and my wife also has a job. So when we put together our income we see that we both have the capacity to provide for our family, therefore no one can isolate me. There are various things causing someone to be put in isolation and the major reason is when one [a man] is dependant which is not my case because I have the capacity to provide for my family … (Rwanda, 45 years, Participant 6)

This narrative sheds light on how earning an income and financial independence not only protects men with disabilities from disrespect, stigma, and discrimination but also enables them to have power in their families and access the idealized form of masculinity in their setting. His assertion that: ‘*The reason why they don’t [discriminate against me] is because in our community I am financially independent’*, evidence this (Rwanda, 45 years, Participant 6).

### Feelings of vulnerability, and lacking recourse and justice

#### Thefts and the lack of formal recourse

Several participants described being taken advantage of by community members, reasoning that this occurred because they lacked physical strength and clout to protect or defend themselves.

Some participants spoke of their assets having been stolen from them and experiencing threats of violence by other men in their communities. They described their limited ‘capabilities’ as a result of their disabilities in terms of physical strength and agility to defend themselves against crime and violence. For example, Participant 4 spoke about his inability to stand up for himself:

I remember a time ago when some people went to steal from my orange nursery, I could not say anything about it [ … ] since I cannot stand and defend myself [ … ] Had it not been [for] my impairment, we would have settled the case with a fight [...] If I confront my suspects, they will end up making trouble for me. I am ‘crippled’ in the leg, whereas they are abled on both legs … Some of them threaten to beat and slap me while others tell me to wait and see. (Ghana, 45 years, Participant 4)

Participant 4 further explained how on one occasion trying to defend himself from a thief put him at risk for further harm. He tried to reprimand the thief, yet the thief used his better physical functioning to threaten him. Hence Participant 4 could neither physically nor verbally defend his property and had his scarce resources taken from him (loss of opportunity), and he had few other opportunities to defend his property (police or community support):

My disability makes me sad, it pains me a lot. Secondly, I feel sad about how my disability limits me; even if someone steals from me, I cannot confront or challenge the person … . The way the thief behaved towards me when I confronted him gave me a reason to believe he was taking advantage of my disability to cheat me [ … ] He threatened to slap and beat me after confronting him of stealing something [oranges] of mine. I was scared. (Ghana, 45 years, Participant 4)

Similarly, Participant 7 claimed he did not get recourse for having his cassava crops stolen:

...last night they [thieves] stole my cassava; what do I do?” … he [man he reports to] would respond to you that: “at least for you, you even had it! They did well to steal from you!” So instead of being laughed at [for trying to retaliate and get beaten], you’d better calm down. (Rwanda, age unknown, Participant 7)

Narrating a similar experience, Participant 8 from Rwanda expressed that men with disabilities have limited ‘power’ to ensure they are respected and not taken advantage of by other men. He recounted how he had lent money to some men in his community but was never repaid. He surmised that these ‘*men knew I did not have the physical strength to demand it back’*. (Rwanda, 55 years, Participant 8).

#### Men’s inability to physically defend themselves

While the majority of the participants did not experience physical violence, their fear and sense of vulnerability were exacerbated by continued verbal threats of violence, mainly from men without disabilities in their communities. Men with disabilities saw their vulnerability and discrimination related to their disability and loss of physical strength as undermining their ability to defend themselves. Participant 4 from Ghana asserted:

If I was an abled person, people like them [men in his community] would not be able to talk to me in such a manner [ … ] Time and time again people talk to me arrogantly and this gets me angry. Though I am a quick-tempered person, my impairment does not allow me to take any action against such people even if I want to [ … ] some of them threaten to beat and slap me [ … ] (Ghana, 45 years, Participant 4)

Although most men did not report any physical violence, in South Africa, Participant 3 shared experiences of women physically assaulting or and sexually harassing him: ‘*Some women undermine me and say I do not have a wife. They also pull my wheelchair and try to take off my clothes and expose my private part*’. (South Africa, 29 years, Participant 3).

In South Africa, a country with particularly high rates of interpersonal violence, our data suggest that some participants’ vulnerability to violence was amplified by their disability. For instance, Participant 9 spoke of other men taking ‘*advantage of the fact that I live with this condition*’ (South Africa, 40 years, Participant 9). Likewise, Participant 2 perceived that his disability compounded his already unsafe environments and feelings of insecurity: ‘*Because they [able-bodied men] see you have a disability, that you can not run away, they can mug you, especially these ‘nyaope’ (local drug) boys, and the murderers will come to you first*. (South Africa, 23 years, Participant 2).

However, participants’ sense of vulnerability to violence was evident across the three settings, as Participant 4 also alluded to in Ghana:

We [people with disabilities] are marginalized in a way hence it has paved a way for others to abuse us, we have no power to protect ourselves. Secondly, we are not physically strong to protect ourselves like the non-disabled people, assuming there is war in the community, a disabled person will not be able to fight his/her way through. People will abuse you. (Ghana, 45 years, Participant 4)

Emphasizing the limitations his disability had on his performance of masculinity, Participant 4 expressed reluctance in engaging in criminal activities like many other men in his community because he was more likely to get caught:

My fear is going to do something secretive or being involved with a group who will go and steal and be chased since I cannot run due to my disability (Ghana, 45 years, Participant 4).

#### Harmful alcohol use

Across all settings, harmful use of alcohol was described as a common male activity. Yet, some participants talked about having reduced their intake of alcohol or chosen safe places to drink to decrease their risk of being violated. For example, Participant 10 from Rwanda mentioned that he stopped drinking with other men in his community, for fear of being victimized when drunk:

… the way I prevented myself from violence, especially that people tried several times to do violence on me because they thought they were stronger than me, the first thing that I did was avoiding to be drunk with them. When they get drunk, they showed a sign of lack of respect because I am “Kajoritie” [ … ] I can’t even lie to you, it happened that they beat me, so the first thing that I did is not to be drunk with them. (Rwanda, 39 years, Participant 10)

In all three settings, drinking with other men was identified as an important social and bonding activity to achieve masculine status and not being able to do so meant that men with disabilities were excluded from participating in a common male activity–which in turn could call into question their attainment of idealized masculinity.

### Provider role, procreation, and decision-making

‘In Akan^2^ culture, … a man is a male figure who takes care of his family by working hard to make sure the family is financially sound’ (Ghana, 62, Participant 11). Across all three settings, material provision for ones’ family was identified as an important marker of masculinity. Yet, some participants described how their impairments, experiences of stigma, and discrimination restricted their employment and or income-generating opportunities and therefore their ability to provide: ‘*They [people] are judgmental. They ask me why I am at work when I am using crutches’*. (Participant 5, 29, South Africa).

Employment was an aspired capability for these men rather than relying on hand-outs from other people or their partners:

Some people would suggest that I go to the traffic lights and make money off my disability. I tell them that it’s not what I would like to do, I want to run my own business. (South Africa, 28 years, Participant 12)

Participant 10 from Rwanda described how his inability to provide for his family led to arguments with his wife, in which she would point out his lack of income and compare it to her ability to provide, making him feel as being seen as “*useless*”:

There is a time when – for example when we need something that we do not have, she [his wife] would tell me: “you are the one responsible for providing for the family and you are not able to. How will we live? [ … ] It used to happen for her to be aggressive towards me, she used to show me that she is the one to do a lot [ … ] what I think about her is that she thinks that I am useless [ … ] You understand that the fact that we were married, that I didn’t have a job and that I had a disability- that was the problem … (Rwanda, 39 years, Participant 10)

As evident in Participant 10’s extract, the dominant norm in Rwanda is for men to be financial providers and sole or primary breadwinners, and if unable to do so, they can feel stuck and be socially undermined [29].

Inability to provide financially also had consequences for participants’ decision-making power within their households, and this was more pronounced in Ghana. In some instances, women fulfilled a provider role, because due to their impairments, men were largely deprived of the opportunity to work. This made some men feel that their dominance/power over their partner and control over decision-making in the household were undermined, both of which were key expectations linked to idealized masculinities:

The man has more votes than a woman in decision-making. This is because the man provides money for the upkeep of the home. The man is the one who bears the financial upkeep of the household … if the housekeeping money is from the woman’s pocket, then the woman will think she has higher votes than the man in decision-making (Ghana, 45 years, Participant 4)

While in all settings the most important marker of ‘successful’ masculinity was the ability to provide financially, a few men noted how they felt that their disability had less negative social implications (less stigma) if they were married or able to procreate or impregnate a woman. Participant 13’s assertion that: ‘ *My situation is okay because at least I am able to have children*’ (Ghana, 51 years, Participant 13), evidences this. Similarly, in Rwanda, Participant 14 asserted that despite continued discrimination by other community members, he felt he was recognized by his wife for being able to sustain a marriage for many years and performing what is expected of him in his home and marriage. In his own words:

There are still some people who still have the mentality that people with disabilities are of no importance. On [the] contrary, my wife has already noticed my importance because it has been 17 years that we are together! … I do what I can do with my one arm. (Rwanda, age unknown, Participant 14)

Similarly, some participants who had limited opportunities to fulfil their provider role–cared for children in the home while their partner went out to work. This signalled a shift in roles and was appreciated by some participants and their partners.

It is because I can’t go anywhere. I babysit my children in the house in case their mother is not around … I like taking care of the children in the house and directing them on how to live a better life so that they won’t go wayward when they grow up. (Ghana, 62, Participant 11)

For some participants like Participant 15 from Rwanda, this rethinking was initiated after attending the Indashyikirwa couples curriculum. Through this, he discovered new opportunities for himself and made more of an effort to engage in household duties – which are often assumed to be ‘*women’s work*’. This was appreciated by his partner:

I used to neglect some work so she would tell me: “will you only stay sitting? Why can’t you try to do some small tasks? She had a feeling that I abandoned her alone in doing everything. Since we received training from RWAMREC, I now do some household tasks therefore she notices that I also try. (Rwanda, age unknown, Participant 15)

#### Feelings of inadequacy, reliance, and loss of virility

The data revealed that impairments and disability stigma impacted sexual relationships, with some participants expressing anxieties over not being desirable to attract an intimate partner because of their disability: ‘*I have a fear if I go to someone, they will not accept me. They would say I am different and that I am disabled’*. (South Africa, age 23 years, Participant 2).

Similarly, Participant 5 shared that his partner often told him he was ‘*worthless*,’ and that she was the only woman who cared for him: *‘ She [partner] also convinced me I was worthless and that’s why everyone avoided me, and that she was the only one who cared about me’*. (South Africa, 29 years, Participant 5).

Participants who acquired a physical impairment later in life, after marriage, or whose impairments had worsened over time reported the frustration of not being able to achieve adequate sexual performance as they had been able to do previously. There was a sense of loss including losses of potential romantic partnerships and loss of sexual prowess and capacity to fulfil expectations of sexual intimacy and garner love and respect from existing partners. The traditional perception of virility as a marker of a ‘real man’, the potential focus on penetrative sex, and lack of alternative methods to achieve partner satisfaction, hence the perceived loss of ability to satisfy the partner left men like Participant 13 feeling worthless:

Sometimes I feel bad when she becomes angry, she gets angry when I can’t work longer at the farm and when I also don’t satisfy her in bed. I used to go three rounds but now I get tired after the first round … I was not disabled when we got married, but these days I can’t satisfy her in bed like I used to do, I don’t have the stamina I used to have, and I also don’t have money these days. (Ghana, 51 years, Participant 13)

Dominant gender norms often dictate that women comply with their spouse’s sexual desires and requests. Our data show how Participant 13 felt disappointed and angered by his partners’ refusal to have sex with him. He felt her behavior was in retaliation to him not contributing to the household finances and bringing in money.

Sometimes she doesn’t cook for me and also she denies me sex...I feel bad about it because I believe money shouldn’t stop anyone from doing her obligation and duties as a wife, sometimes I feel like cheating on her. (Ghana, 51 years, Participant 13)

While Participant 13’s quote shows that some men ascribed to conservative and rigid gender norms and aspired to be dominant in their intimate relationships, a contrasting experience was given by Participant 5 who reported that his former girlfriend was controlling and her condescending behaviour towards him led to him having a physical fight with her. His case also shows that he does not believe authorities such as the police would help him:

I stayed with my girlfriend. She took advantage that I was disabled. I had to do everything she asked. Because it was like I did not have a life. I did most of the washing and cooking. She was controlling. We ended up fighting [physically] to a point where it [seemed like] I was violent. Even when I could have gone to the police station, they would be on her side. (South Africa, 29 years, Participant 5)

Some participants agreed with the view held by many men in these settings that if their female partners do not acquiesce to sex, they have a right to have other concurrent sexual partners. However, as Participant 13 claimed, many participants opined that it was difficult for them to have other sexual partners as they were typically found less desirable by women, had reduced stamina for sex, and/or lacked the money to give other partners:

I will feel fine if I get my needs elsewhere because she is not performing her duties … For now, I do not have that right [ … ] I would have cheated on her if it was before my impairment … My energy/stamina to have sex has reduced. That is the reason why I will not cheat on her. (Ghana, 51 years, Participant 13)

These data suggest that physical disability may hinder men from coercing female partners sexually, even if they would have wanted to. This is best illustrated by Participant 10, from Rwanda, who when asked whether he had forced sex on his paper replied: *How can I do it [sex] by force when I am “Kajorite”* 3*? I can’t even think about it’*. (Rwanda, 39 years, Participant 10).

Moreover, for many of the participants, the stamina to perform multiple rounds of sex was unattainable, leaving the participants questioning their sexual prowess or virility, an essential characteristic of idealized masculinity identified in these settings. Against the background of feeling threatened, powerless and marginalized, men with disabilities experienced feelings of unworthiness and seemed to lack knowledge about alternative ways of engaging their partners in their relationship and sex life.

## Discussion

In this study, we explored whether and how physical disability intersects with constructions of masculinities and violence perpetration and experiences among adult African men with physical disabilities in Ghana, Rwanda, and South Africa. Broadly, our findings suggest that men with physical disabilities tended to measure themselves – and are measured by others–according to dominant norms and markers of idealized masculinity, while at the same time experiencing deprivation of the opportunities to fulfil these dominant notions of masculinity (lack of capability). Similar to Barrett [19] and Schuttleworth’s [18] papers deriving from resource-rich settings, disability is socially constructed as “weak” and in contrast with dominant notions of “masculinities” in the three study settings. In line with Cornell’s view [7], inenqualities among men were observed in all three countries; while men in all three countries may find it difficult to be a provider and fullfil local notions of masculities (resourse poor context), men with disabilities experienced greater oppression than their peers without disabilities, because they could not fullfil the cultural notions of masculinity as being ‘physically strong’ and providers in their homes.

Men in this study found their sense of masculinity challenged daily in different settings, be it in the fields and gardens, in taverns or the street, village meetings, by able-bodied men, their intimate partners, or through self-reflection, impacting their self-presentation, body image, sexuality, and relationships. However, for some participants the lack of opportunities to attain some markers of the idealized masculinities (e.g. being the family provider) and certain practices associated with these masculinities (e.g. alcohol abuse, multiple partnerships, and sexual coercion) afforded them the space to reimagine alternative masculinities, change and take up caring roles such as child care or practice more safe behaviours such as reduced alcohol drinking and quarrelling with other men, and not coerce their partners to have sex. Barrett [19] reveals how dilemmas related to masculinities are retained, negotiated, or dismissed by men with disabilities in resource-rich settings in multiple ways and according to different impairment types and severity; age, and contexts; and particularly concerning men’s access to resources.

Our findings suggest that men with physical disabilities in these three African countries experience many deprivations of opportunities and alongside this, capabilities. Our findings seem to indicate that many men lost access to society (i.e. were socially excluded) as well as lost access to valorized gender roles including being providers for the family, ‘respected persons’, and decision-makers. Our findings suggest that men who had acquired a disability later in life experienced exaggerated isolation, as their previous social networks and interactions became narrowed due to their new impairment, loss of income, and environmental. Similar expressions of exclusion from community activities and restricted decision-making power at community meetings have been made by visually impaired men in Namibia [30] and in many other economic and livelihood focused disability studies in Africa [31–38]. These studies highlight societal stigmatization, exclusion and economic deprivation of persons with disabilities in African counties.

In many contexts in Africa and elsewhere, expectations of physical expressions of the masculine agency are dominant, including the ability of a man to confront injustice, protect his assets and property, ‘value his dignity’ but also be able to participate in physical labour and be sexually active [39]. Our participants recounted restricted ability to assert themselves or seek redress for theft, stating that attempts at doing so resulted in them experiencing threats of violence or public ridicule. They linked this explicitly to being taken advantage of and humiliated because of their disability. Research in South Africa has intimated that for men, violence is often a way of reclaiming dignity and respect [39]. Literature suggests that the assumptions of men with disabilities as weak – and as not able to use masculine-coded violence to respond to humiliation or abuse – may lead men to feel more susceptible to both violence perpetration and victimization [40].

Our findings suggest various themes of marginalization and deprivation of men with disabilties in their communities, including stigma and discrimination, environmental barriers, receiving threats of violence from men without disabilties and other people in the community, and experiencing emotional abuse especially from men without disabilities. Hence contrasting to work with women with disabilities, men with disabilities report threats, verbal and physical abuse by people outside their household and emotional abuse within their housholds and not the same level of sexual and intimate partner violence as women with disabilities have reported in similar studies [41–45].

Overall, participants did not report any severe experiences of IPV and non-partner violence – but mostly expressed receiving threats of violence and feeling vulnerable. These men’s experiences differed from those of female participants with disabilities from the same study who reported experiences of IPV and non-partner violence [23]. This suggests that men with disabilities may not share the risk factors for, and extent of violence that women with disabilities in the Global South often face [46]. Yet, their experiences shed light on the vulnerabilities of men with physical disabilities living in the Global South. This study suggests that the asumptions of men with disabilities as ‘weak’ is problematic as it increases the perception that they cannot defend themselves and are therefore an easy target for violence, abuse or harassment, yet this may also distract from the fact that men with disabilities can support the notion of violence against female parterns as acceptable and be potential perpetrators of violence against their female partners [19].

In South Africa, Rwanda, Ghana, masculine respect and authority is also largely underpinned by providing economically for partners and families [47, 48]. However, masculine norms that stress the primacy of men’s work which in rural areas and informal settlements tends to be manual labour, are in itself masculine-coded if the available work request physical labour. Mobility limitations restrict a person’s ability to conduct some physical activities, and thus be self-reliant in spaces where physicial labour is the prime opportunity to work.

In our study, participants reported that their productivity and providing for their families was undermined by a lack of mobility, dexterity, and physical stamina, as well as discrimination in the labour market and lack of accessible work opportunities. Hence using the capability approach – these men lacked practical opportunities [17]. As shown in other studies [49], lack of practical opportunties often caused discord with intimate partners and could fuel poor mental health, including lack of self-respect or self-esteem due to their inability to meet social expectations of masculinity, be it as spouses, breadwinners and fathers. Achieving provider role in contexts of deprivation is difficult in general, men in general may feel socially undermined [50], and these challenges may be exacerbated for men living with disabilities.

Recent evidence from South Africa suggests that men with disabilities experience higher rates of unemployment, underemployment, precarious employment and poverty, and lower labour force participation rates and incomes, than men without disabilities – although, significantly, men with disabilities continue to accrue privilege over women with disabilities [51, 52]. Similar findings have been reported across Africa, revealing that persons with disabilities experience multidimensional poverty with women with disabilities experiencing more deprivation then men with disabilities [53].

The performance of masculine gender identities is further bound up with the realm of sexuality, and in particular social expectations that encourage men to prove their manhood through (hetero) sexual conquest [7]. However, Nolan [54] describes a sense of loss of masculinity among men who acquire impairments as adults, which was also evidenced in our data. Sexual endurance and performance are often perceived as a traditional trademark of a “real man”, yet, respondents expressed their anxieties around sexual performance, such as not being able to “go three rounds”, while lacking ideas on how to engage in sexual activity despite their mobility limitations. This study findings are in line with previous literature which has contended that conformity to gender norms stressing the importance of men’s (penetrative) sexual capacity over other romantic or erotic activities exacerbates the lack of confidence of men with disabilities to satisfy their partners and increases the focus on their disability-related changes in sexual potency/functioning [55]. Furthermore, loss of sexual stamina also meant participants felt unable to engage in multiple concurrent partner relationships, even though they felt entitled to do so if their partner didn’t satisfy them enough. In a similar vein, other research has found that men with disabilities are less likely to report engaging in sexual risk behaviours, such as multiple concurrent partners [56]. Indeed research on HIV and disability has revealed that women with disabilities are at particular risk of exposure to HIV because of their exposure to sexual violence, exploitation and transactional sexual activities [57, 58].

Our findings suggest that some men felt their partners’ refusal to have sex with them meant that their desire for sexual connections was unfulfilled, which the men themselves often framed as ‘unmet needs’. Published literature suggests that such attitudes reflect notions of male sexual entitlement, often associated with masculinities in sub-Saharan Africa and elsewhere [2, 59]. In many settings, marriage is interpreted by both men and women as granting men unencumbered sexual access to their wives [60]. The opinions of our participants echo those of men without disabilities. Research in Ghana and Rwanda with men without disabilities showed that norms around men’s entitlement to sex were strong and based on notions that “marriage entitles men to unconstrained sexual access to their spouse; men alone should initiate and decide the terms of sex, and women should never refuse sex with their husband or partner” [48, 61].

Other studies have shown that because of their impairments, and stigma around disability, men with disabilities may experience nuanced and complicated forms of community-level violence that might not be immediately recognizable to outsiders [62].

While our findings seem to indicate that some men were able to participate in community activities and work, and dismissed disability stigma (not internalize it), others had fewer opportunities to fulfil constructions of masculinities within their contexts. This study suggests that there was significant diversity in how men managed and perceived their limits to masculinity, often shaped by the severity, onset, and type of impairment they had. Our findings support Charmaz’ findings that men acquiring disabilities may have increased difficulties in “preserving [the] self to maintain a sense of coherence while experiencing loss and change” [63]. Furthermore, exacerbating and underscoring their feelings of not living up to dominant expected performances and markers of idealized masculinity, many men in our study reported being repeatedly challenged for these ‘shortcomings’ by other men and women in their communities.

In summary, some men’s aspirations to be respected and welcomed in their communities, to be providers so they could establish their decision making power within their households, and valuing sexual prowess suggest that they sought inclusion to the idealized masculinity which was hegemonic in these settings instead of seeking inclusion as an equal member of society acknowledging the equality between men and women. Notwithstanding, however, our findings suggest that if impairments did not deprive men of the roles and opportunities (capabilities) described here, they may have felt less isolated and depressed and experienced less stigma.

### Implications for health programmes

The participants’ narratives of social exclusion and decreased participation in their community activities may have implications for their involvement in health and development programmes. Specifically, this highlights the need for programmmers who work with men in these settings to reduce their exposure to violence and potential stigmatization and shaming (e.g. through ridicule), use of violence, to ensure their inclusion in violence prevention and gender transformative programmes. The men with disabilities inclusion in such programme is critical as their engagement in such programmes may in itself help address the stigma and discrimination of men with disabilities.

Linked to this is the importance of violence prevention and gender-transformative programmes to challenge harmful norms of masculinity through working with men with disabilities to critically reflect on different ways to attain status as men, and the benefits of non-violence, more gender-equitable, and non-violent masculinity. This points to the need for programmes to support men with disabilities to re-shape their masculinities to take pride in engaging in caring roles, equal and supportive relationships and be involved parents to their children.

Such work is especially important as men with disabilities in our sample aspired to be included in the idealized masculinities that were circulating in their settings. Our findings seem to indicate that most men had gender-inequitable attitudes exhibited by their desire to control their female partners and be dominant in intimate relationships. As such, engaging men with disabilities to re-frame their masculinities could also generate self-esteem, healthy sexual relationships and help combat internal and societal stigma.

### Recommendation for future research

Further research is needed to establish how access to employment for men with disabilities shape their experiences of IPV in their homes and stigma and violence in their communities. Also, qualitative research is needed to examine how men with disabilities conceptualize masculine authority within intimate relationships and IPV, and also how couples could be supported to discover new opportunities to live their sexuality despite a disability that does not enable sexual penetration and stamina. Furthermore, future studies would do well to understand how the spectrum of impairment impacts men’s experiences of gender and IPV differently, considering that men with severe disabilities, and men with cognitive disabilities, may be at higher risk of experiencing violence, and are less likely to access GBV prevention interventions [23]. Last, there is a need for larger studies that can enable exploration of differences or similariles between men with disabilities that may exist becuase on their place of origin.

### Limitations

The study may have limitations resulting from the data collection method used in the three study settings. In Rwanda the interviews were conducted by a female researcher whereas in South Africa and Ghana were conducted by male interviewers. The gender of the interviewers may have influenced how men responded to the interview situation and the questions asked. For instance, it is likely that being interviewed by a female researcher about issues related to disability, violence, and health may have reduced some men’s openness to talk about personal information. On the other hand, some men may have found the interview space nurturing and safer when speaking to a female researcher compared to male interviewers.

Also, there is a potential for social desirability bias that may have impacted the views expressed by men in this sample. Men may have over-reported “good behaviour” or “vulnerability” and under-reported undesirable behaviour. We tried to mitigate this by emphasizing confidentiality, and the use of repeat interviews may have been useful for building rapport and supporting openness.

While some participants reported experiencing emotional, sexual, and physical violence from community members and intimate partners, none of them reported having perpetrated violence. Men in this study may have not reported violence perpetration due to normative restrictions against men admitting to experiences of coercion or violence.

## Conclusions

Our findings suggest that men’s physical disability had important implications for their construction of masculinities, exposure to violence, especially emotional violence by able-bodied men, and inability to access aspects of idealized masculinity which they valorized. These findings are important as an empirical base for the conceptualization of context-specific prevention interventions that target disability and gender-based violence and gender-transformative programmes that aim to also engage men with disabilities.

The dearth of data regarding the experiences of victimization and perpetration of violence among men with disabilities in the Global South means that the scholarship on this topic remains limited especially around how men with a disability could be meaningfully involved in disability specific, violence prevention and gender-transformative programming through redefining notions of masculinity to include roles and activities that are less dependent on physical strength, dominance and mobility (e.g. caring for or teaching children, housework, community leaders). Such understanding can also help prevention programmes consider how to be more inclusive of men and women with disabilities.

The absence of men with disabilities in existing violence interventions is likely to maintain their marginalization, lack of opportunities, and exposure to various forms of violence. Further work is required to promote more inclusive programming–being mainstream and targeted to men with disabilities in Africa and other countries in the Global South.

## Data Availability

All data from interviews with participants are presented in the manuscript.

## Abbreviations

IPV: Intimate partner violence
IDI: In-depth interview

## Footnotes

1
https://whatworks.co.za/

2 In Ghana, the Akan are the dominant tribal group.

3 In Kinyarwanda the term *Kajorite* is a pejorative name they call people with physical disabilities. Most of the time it is a disability with arms or/and legs. Another word used is *ikimuga* meaning “broken pot”, and also *Kajisho* which means: one small eye/one-eyed person
